# *Staphylococcus aureus* pigmentation is not controlled by Hfq

**DOI:** 10.1186/s13104-020-4934-4

**Published:** 2020-02-07

**Authors:** Wenfeng Liu, Pierre Boudry, Chantal Bohn, Philippe Bouloc

**Affiliations:** Université Paris-Saclay, CEA, CNRS, Institute for Integrative Biology of the Cell (I2BC), 91198 Gif-sur-Yvette, France

**Keywords:** *Staphylococcus aureus*, Hfq, Pigmentation, Staphyloxanthin, Regulation

## Abstract

**Objective:**

The golden color of *Staphylococcus aureus* is due to the synthesis of carotenoid pigments. In Gram-negative bacteria, Hfq is a global posttranscriptional regulator, but its function in *S. aureus* remains obscure. The absence of Hfq in *S. aureus* was reported to correlate with production of carotenoid pigment leading to the conclusion that Hfq was a negative regulator of the yellow color. However, we reported the construction of *hfq* mutants in several *S. aureus* strains and never noticed any color change; we therefore revisited the question of Hfq implication in *S. aureus* pigmentation.

**Results:**

The absence or accumulation of Hfq does not affect *S. aureus* pigmentation.

## Introduction

*Staphylococcus aureus* is a major pathogen responsible for numerous diseases from minor skin infection to septicemia, affecting humans and other animals. Its name “*aureus*” comes from the golden color of strains that express carotenoid pigments [1]. These pigments contribute to oxidative stress and neutrophil resistance, and virulence [2]. The carotenoid biosynthetic operon (*crtMNOPQ*) leading to the synthesis of staphyloxanthin is regulated by σ^B^ [3, 4], an alternative σ factor that also controls a large number of general stress genes. σ^B^ activity depends on RsbU, its positive regulator [5, 6]. Numerous strains, including the *S. aureus* model NCTC8325, have *rsbU* mutations that prevent σ^B^ activity and *crt* operon expression, such that colonies are white. In addition, mutations in 37 genes were shown to result in the loss of a yellow pigmentation [5, 7].

Hfq is an RNA chaperone needed for activity of numerous regulatory RNAs in Gram-negative bacteria [8]. However, its role in Gram-positive bacteria, with the exception of *Clostridium difficile* [9], remains enigmatic [10]. Hfq functionality from different species is often tested by interspecies complementation tests. However, expression of *hfq* genes from Gram-positive bacteria *S. aureus* and *Bacillus subtilis* in *Salmonella* could not compensate the absence of endogenous *hfq*, indicating a functional difference between Gram positive and negative Hfq [11, 12].

We previously compared phenotypes of *S. aureus hfq* mutants with their isogenic parental strains and observed no detectable difference associated with the absence of Hfq in the tested conditions [13]. However, our results were partly challenged by a publication reporting that carotenoid pigment production was increased in *hfq*-negative strains [14]. Here we use nine different *S. aureus* strains to show that Hfq absence or overexpression has no effect on pigment expression.

## Main text

### Methods

#### Bacterial strains, plasmids and growth conditions

Bacterial strains, plasmids and primers used in this study are listed in Table 1. Allelic replacements of *hfq*^+^ by Δ*hfq*::cat were either performed by ϕ11-phage mediated transduction using RN4220 *hfq*::cat as a donor strain or by homologous recombination using pMADΔhfq::cat [13, 15]. The Δ*hfq*::*cat* deletion in SAPHB5 was verified by Southern blot and subsequent Δ*hfq*::*cat* transductants were verified by PCR as described [13].

**Table 1:** *Staphylococcus aureus* strains, plasmids and primer used for this study

Strain name	Key features	Reference or construction
RN4220	Transformable by DNA from *E. coli*	[25]
SAPhB5	RN4220 Δ*hfq*::*cat*	[13]
NCTC8325	Clinical isolate	[26]
SAPhB224	NCTC8325 Δ*hfq*::*cat*	NCTC8325 + ϕ11(SAPhB5)
NCTC8325-4	NCTC8325 Δϕ11 Δϕ12 Δϕ13	[27]
SAPhB197	NCTC8325-4 Δ*hfq*::*cat*	NCTC8325-4 + ϕ11(SAPhB5)
RN6390	NCTC 8325-4 ϕ6390	[28]
SAPhB22	RN6390 Δ*hfq*::*cat*	[13]
HG001	NCTC8325 *rsbU* repaired	[22]
SAPhB199	HG001 Δ*hfq*::*cat*	HG001 + ϕ11(SAPhB5)
HG002	NCTC8325 *tcaR* repaired	[22]
SAPhB201	HG002 Δ*hfq*::*cat*	HG002 + ϕ11(SAPhB5)
HG003	NCTC8325 *rsbU* and *tcaR* repaired	[22]
SAPhB203	HG003 Δ*hfq*::*cat*	HG003 + ϕ11(SAPhB5)
COL	Methicillin resistant clinical isolate	[29]
SAPhB16	COL Δ*hfq*::*cat*	[13]
Newman	Clinical isolate	[30]
SAPhB17	Newman Δ*hfq*::*cat*	[13]
SAPhB142	RN4220 pRMC2	RN4220 + pRMC2
SAPhB248	RN4220 pRMC2Hfq	RN4220 + pRMC2Hfq
SAPhB251	RN4220 pRMC2HfqFLAG	RN4220 + pRMC2HfqFLAG
SAPhB233	HG003 pRMC2	HG003 + pRMC2
SAPhB249	HG003 pRMC2Hfq	HG003 + pRMC2Hfq
SAPhB257	HG003 pRMC2HfqFLAG	HG003 + pRMC2HfqFLAG

Plasmid name	Key features	Reference/construction
pRMC2	Anhydrotetracycline (aTc) inducible promoter P_xyl/tetO_	[17]
pRMC2Hfq	*hfq* inducible expression	See “Methods”
pRMC2FLAG	pRMC2 derivative for translational gene fusions with *3xflag* coding sequence	See “Methods”
pRMC2HfqFLAG	*hfq::3xflag* inducible expression	See “Methods”

Primer name	Sequence	Purpose
39	GGGGTACCATGATTGCAAACGAAAAC	*hfq* amplification (with a KpnI site)
49	GGGGAATTCTTATTCTTCACTTTCAGTAGATGC	*hfq* amplification (with an EcoRI site)
856	GGTACCGTTAACAGATCTGAG	pRMC2 amplification
918	GCTTATTTTAATTATACTCTATCAATGATAGAG	pRMC2 and pRMC2FLAG amplifications
858	TCAGATCTGTTAACGGTACCGGAATTAGCTTGCATGGAA	*3xflag* amplification
919	GATAGAGTATAATTAAAATAAGCGAGCTCGACTACAAAGACCA	*3xflag* amplification
865	GACTACAAAGACCATGACGG	pRMC2FLAG amplification
939	GATAGAGTATAATTAAAATAAGCGTAAAAGGAGTCCGACAGATGA	*hfq* amplification for cloning in pRMC2FLAG
940	CCGTCATGGTCTTTGTAGTCTTCTTCACTTTCAGTAGATGCTTG	*hfq* amplification for cloning in pRMC2FLAG

Engineered plasmids were constructed as described [16]. Conditional *hfq* expression was obtained by cloning *hfq* under the xyl/tetO promoter in pRMC2 [17] and pRMC2FLAG (Table 1). pRMC2Hfq allowing *hfq* conditional expression was obtained as follows: pRMC2 and PCR-amplified *hfq* (using primers 39/49 on HG003 DNA) were KpnI-EcoRI digested and ligated together. pRMC2FLAG was engineered for conditional expression of 3xFLAG-tagged proteins as followed: pRMC2 and pSUB11 [18] were PCR-amplified using primers 856/918 and 858/919, respectively. The two resulting products, i.e. pRMC2 and *3xflag* coding sequence, were assembled using the Gibson method [19]. pRMC2HfqFLAG, allowing conditional expression of Hfq::3xFLAG, was obtained as follows: pRMC2FLAG and *hfq* HG003 were PCR-amplified using primers 918/865 and 939/940, respectively. The two resulting products were assembled using the Gibson method.

Bacteria were grown in BHI medium (BD Difco, ref: 237500) at 37 °C under vigorous agitation. BHI solid media were obtained by the addition of Bacto Agar 15 g l^−1^ (BD Difco, ref: 214010). For strains containing pRMC2 and derivatives, chloramphenicol (Sigma-Aldrich, ref: C0378) 5 µg ml^−1^ was added to media. Expression from pRMC2 and derivatives was achieved by anhydrotetracycline (aTc, Chemodex, ref: CDX-A0197-M500) 250 ng ml^−1^ addition to growth media.

#### Protein extraction, Western blotting and staphyloxanthin spectral measurement

Overnight cultures were diluted 1000 times in fresh medium. After 3 h, aTc was added. 10 min and 30 min later, cells were harvested by centrifugation (16,000*g* for 2 min), resuspended in 400 µl Tris HCl buffer (50 mM, pH 6.8) and lysed using a FastPrep (3 cycles of 45 s at 6.5 m s^−1^). Cell debris was removed by centrifugation (16,000*g* for 10 min). Protein concentration was determined by Bradford assays [20]. For each sample, 3 μg of protein extract was separated on a polyacrylamide gel (Blot™ 4–12% Bis–Tris Plus, Invitrogen, ref: NW04122BOX). After electrophoresis, proteins were transferred to a polyvinylidene fluoride membrane (iBlot 2 PVDF Mini Stacks, Invitrogen ref: IB24002). For blotting and washing, an iBind™ Flex Western System (ref: SLF2000S) was used according to supplier’s instructions. Membranes were probed with the primary polyclonal ANTI-FLAG antibody produced in rabbit (Sigma, ref: F-7425) at a 1/15,000 dilution. A rabbit secondary antibody conjugated to horseradish peroxidase (Advansta, ref: R-05072-500) was used at a 1/25,000 dilution. Bioluminescent signal was detected with the WesternBright™ ECL-spray (Advansta, ref: K-12040-D50) using a digital camera (ImageQuant™ 350, GE Healthcare).

The *S. aureus* pigments were extracted as described [21]. In brief, strains were grown in BHI under vigorous agitation for 24 h. Cells were harvested by centrifugation, the pellet was rinsed twice with sterile water and pigments were extracted by methanol. Absorbance between 330 and 550 nm was measured on a microplate reader (CLARIOstar BMG LABTECH).

### Results

#### The absence of Hfq does not alter *S. aureus* pigmentation

In 2010, Liu et al. reported that “deletion of *hfq* gene in *S. aureus* 8325-4 can increase the surface carotenoid pigments” [14]. Their work was performed using an allele called *Δhfq*-*8325* in which the *hfq* coding sequence was replaced by a kanamycin cassette. The *hfq* chromosomal deletion was constructed in strain RN4220 and then transduced into NCTC8325-4, RN6390, COL and ATCC25923 by phage ϕ11. We constructed a similar *hfq* deletion in RN4220, except that the *hfq* coding sequence was replaced by a chloramphenicol resistant gene (Δ*hfq*::cat); this allele was transduced into RN6390, COL and Newman by ϕ11-phage mediated transduction [13]. Note that RN4220, RN6390 and COL strains were used for both studies. As we did not notice a change of color when the Δ*hfq*::cat allele was introduced into these strains, this information was not reported [13]. In view of the previous report, we focused this work on the possibility that Hfq could affect *S. aureus* pigment expression.

**Fig. 1:**
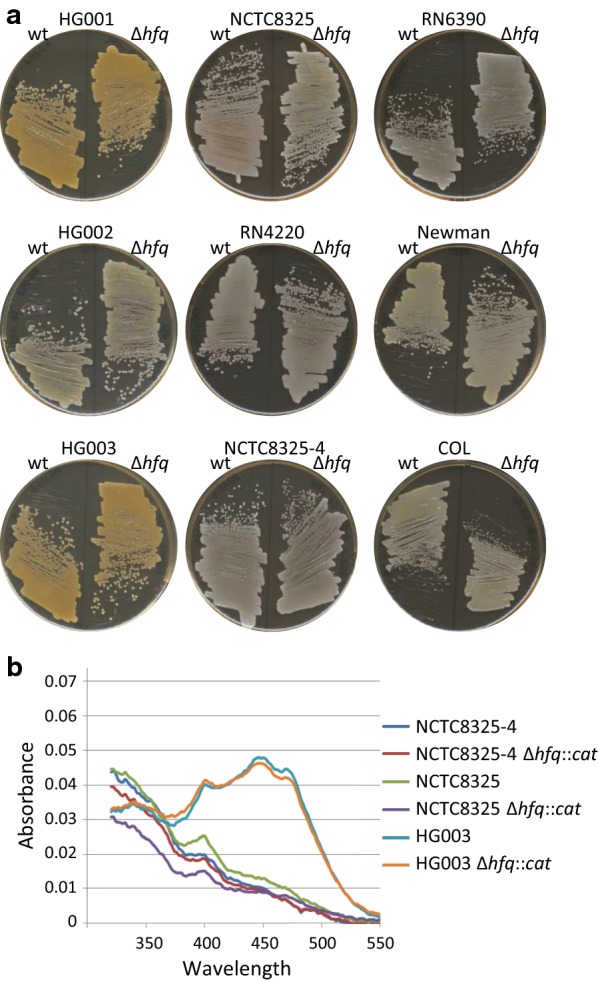
Absence of Hfq does not affect *S. aureus* pigmentation. The indicated strains were grown overnight in BHI and then **a** streaked on BHI agar or **b** assayed for spectral profiles as described [21]

NCTC8325 isolated in 1960 from a sepsis patient is the progenitor of numerous strains including NCTC8325-4 (cured of three prophages) which itself gave RN6390 and RN4220 [22]. As these descendants were mutagenized, they carry several mutations that may affect their phenotypes. NCTC8325 has a deletion of 11 bp in *rsbU* and a point mutation in *tcaR*. The derivatives HG001 (*rsbU* restored), HG002 (*tcaR* restored), HG003 (*rsbU* and *tcaR* restored) were constructed to perform physiological studies in a non-mutagenized background [22]. All these NCTC8325 derived strains, except HG001 and HG003 (which have a functional σ^B^ factor), give rise to white colonies (Fig. 1). In addition to those reported [13], we constructed Δ*hfq*::cat derivatives in NCTC8325, NCTC8325-4, HG001, HG002 and HG003 (Table 1). In contrast to results reported in Liu et al., deletion of the *hfq* gene in all tested strain backgrounds had no effect on pigmentation (Fig. 1a). Note that COL, Newman are not NCTC8325 derivatives.

Spectral profiles highlighting *S. aureus* carotenoid production were determined as described [21] for three strains and their *hfq* derivatives after growth for 24 h in BHI. HG003 and HG003 Δ*hfq*::*cat* gave equivalent profiles with three pics characteristic of carotenoid production. In contrast, NCTC8325-4 and RN1 had spectra characteristic of no or very little carotenoid production. As expected from our visual observation (Fig. 1a), the spectra of Δ*hfq* derivatives did not differ from those of their respective parental strains (Fig. 1b).

#### Hfq overexpression does not alter *S. aureus* pigmentation

**Fig. 2:**
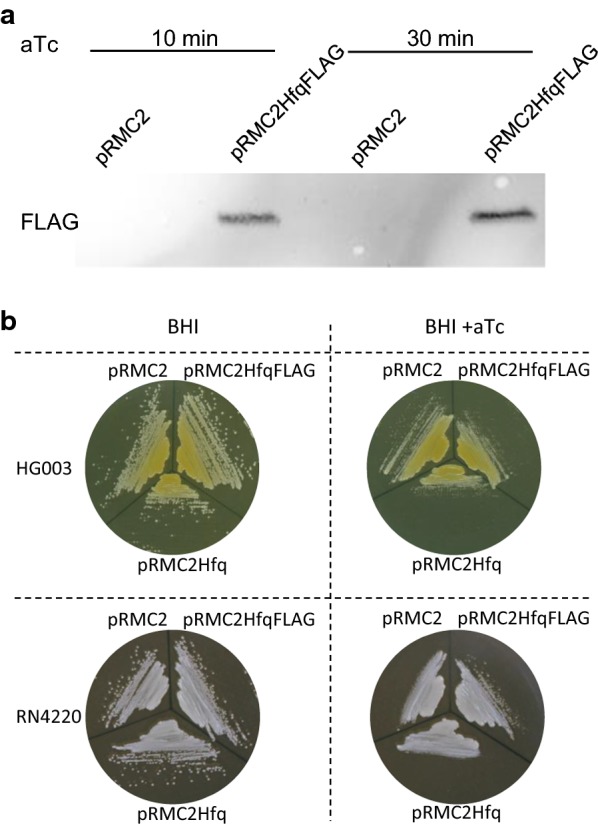
Accumulation of Hfq does not affect *S. aureus* pigmentation. The indicated strains were grown in BHI supplemented with chloramphenicol. They were then **a** assayed by Western blotting for *hfq*::*3xflag* expression (see “Methods”) and **b** streaked on BHI agar supplemented with aTc when indicated

In the above-described strains, *hfq* is possibly poorly expressed, in which case *hfq* deletions would not lead to detectable phenotypes. We therefore tested the effects of an inducible Hfq expression system on pigment production. If the absence of Hfq leads to yellow colonies as proposed [14], the presence of Hfq could lower pigment production and lead to white colonies. To address this point, *hfq* was cloned under the control of the P_xyl/tetO_ promoter in multi-copy plasmid pRMC2 [17] leading to pRMC2Hfq. *hfq* expression in strains harboring pRMC2Hfq was induced upon aTc addition to media. To confirm that P_xyl/tetO_ was effectively driving *hfq* expression, a pRMC2Hfq derivative was engineered harboring a *3xflag* sequence inserted in frame at the end of the *hfq* open reading frame. The resulting plasmid, pRMC2HfqFLAG is a proxy for expression from pRMC2Hfq. HG003 was transformed with pRMC2, pRMC2Hfq and pRMC2HfqFLAG. The protein Hfq::3xFLAG was detected upon aTc induction by western blotting using FLAG antibodies (Fig. 2a). We inferred from this result that addition of aTc to strains harboring pRMC2Hfq lead to Hfq synthesis. The RN4220 white and HG003 yellow colors were not affected by the presence of either pRMC2, pRMC2Hfq or pRMC2HfqFLAG and remained identical upon aTc addition to growth medium (Fig. 2b).

### Conclusion

Our results show that neither the absence, nor the accumulation of Hfq affects pigmentation of *S. aureus*: Hfq does not appear to regulate staphyloxanthin synthesis. Our conclusions are supported by Tarrant PhD dissertation showing an NCTC8325 *hfq* mutant that remained unpigmented [23]. Of note, *Pseudomonas aeruginosa* reportedly induces pigment production of a non-pigmented phenotypic variant of *S. aureus*, however, this effect was independent of *hfq* transcription [24]. In addition, color variation in USA300 strain was screened in a genome-wide transposon mutant library, and the *hfq* inactivation was not reported to affect *S. aureus* pigmentation [7].

While the *hfq* gene is absent in some Firmicutes (e.g. Lactobacillales), it is conserved in all *S. aureus*, suggesting that it plays a crucial function, however not related to pigment expression. The quest to find the Staphylococcal Hfq function remains open.

## Limitations

Our conclusion is in contradiction with Liu et al. results concerning the effect of Hfq on *S. aureus* pigmentation [14]. We cannot rule out that our observation is limited to specific *S. aureus* strains. However, we used an NCTC8325-4 *hfq* derivative similar the one used in the previous study. Furthermore, the present results are strengthened by the construction of *hfq* mutants in numerous *S. aureus* backgrounds. The discrepancy between our and Liu et al. 2010 [14] results, is a possible inadvertent selection of mutants with altered color patterns (as shown in [23]) in the former study.

## Data Availability

All data generated or analyzed during this study are included in this published article. Strains and plasmids are available from the corresponding author on reasonable request.

## Abbreviations

PCR: Polymerase chain reaction
BHI: Brain heart infusion
aTc: Anhydrotetracycline

## References

[1] Marshall JH, Wilmoth GJ (1981). Pigments of *Staphylococcus aureus*, a series of triterpenoid carotenoids. J Bacteriol.

[2] Liu GY, Essex A, Buchanan JT, Datta V, Hoffman HM, Bastian JF, Fierer J, Nizet V (2005). *Staphylococcus aureus* golden pigment impairs neutrophil killing and promotes virulence through its antioxidant activity. J Exp Med.

[3] Bischoff M, Dunman P, Kormanec J, Macapagal D, Murphy E, Mounts W, Berger-Bachi B, Projan S (2004). Microarray-based analysis of the *Staphylococcus aureus* sigmaB regulon. J Bacteriol.

[4] Pelz A, Wieland KP, Putzbach K, Hentschel P, Albert K, Gotz F (2005). Structure and biosynthesis of staphyloxanthin from *Staphylococcus aureus*. J Biol Chem.

[5] Palma M, Cheung AL (2001). sigma(Beta) activity in *Staphylococcus aureus* is controlled by RsbU and an additional factor(s) during bacterial growth. Infect Immun.

[6] Giachino P, Engelmann S, Bischoff M (2001). Sigma(B) activity depends on RsbU in *Staphylococcus aureus*. J Bacteriol.

[7] Fey PD, Endres JL, Yajjala VK, Widhelm TJ, Boissy RJ, Bose JL, Bayles KW (2013). A genetic resource for rapid and comprehensive phenotype screening of nonessential *Staphylococcus aureus* genes. MBio.

[8] Vogel J, Luisi BF (2011). Hfq and its constellation of RNA. Nat Rev Microbiol.

[9] Boudry P, Gracia C, Monot M, Caillet J, Saujet L, Hajnsdorf E, Dupuy B, Martin-Verstraete I, Soutourina O (2014). Pleiotropic role of the RNA chaperone protein Hfq in the human pathogen *Clostridium difficile*. J Bacteriol.

[10] Bouloc P, Repoila F (2016). Fresh layers of RNA-mediated regulation in Gram-positive bacteria. Curr Opin Microbiol.

[11] Rochat T, Bouloc P, Yang Q, Bossi L, Figueroa-Bossi N (2012). Lack of interchangeability of Hfq-like proteins. Biochimie.

[12] Rochat T, Delumeau O, Figueroa-Bossi N, Noirot P, Bossi L, Dervyn E, Bouloc P (2015). Tracking the elusive function of Bacillus subtilis Hfq. PLoS ONE..

[13] Bohn C, Rigoulay C, Bouloc P (2007). No detectable effect of RNA-binding protein Hfq absence in *Staphylococcus aureus*. BMC Microbiol.

[14] Liu Y, Wu N, Dong J, Gao Y, Zhang X, Mu C, Shao N, Yang G (2010). Hfq is a global regulator that controls the pathogenicity of *Staphylococcus aureus*. PLoS ONE..

[15] Le Lam TN, Morvan C, Liu W, Bohn C, Jaszczyszyn Y, Bouloc P (2017). Finding sRNA-associated phenotypes by competition assays: an example with *Staphylococcus aureus*. Methods.

[16] Rochat T, Bohn C, Morvan C, Le Lam TN, Razvi F, Pain A, Toffano-Nioche C, Ponien P, Jacq A, Jacquet E (2018). The conserved regulatory RNA RsaE down-regulates the arginine degradation pathway in *Staphylococcus aureus*. Nucleic Acids Res.

[17] Corrigan RM, Foster TJ (2009). An improved tetracycline-inducible expression vector for *Staphylococcus aureus*. Plasmid.

[18] Uzzau S, Figueroa-Bossi N, Rubino S, Bossi L (2001). Epitope tagging of chromosomal genes in Salmonella. Proc Natl Acad Sci USA.

[19] Gibson DG, Young L, Chuang RY, Venter JC, Hutchison CA, Smith HO (2009). Enzymatic assembly of DNA molecules up to several hundred kilobases. Nat Methods.

[20] Bradford MM (1976). A rapid and sensitive method for the quantitation of microgram quantities of protein utilizing the principle of protein-dye binding. Anal Biochem.

[21] Morikawa K, Maruyama A, Inose Y, Higashide M, Hayashi H, Ohta T (2001). Overexpression of sigma factor, sigma(B), urges *Staphylococcus aureus* to thicken the cell wall and to resist beta-lactams. Biochem Biophys Res Commun.

[22] Herbert S, Ziebandt AK, Ohlsen K, Schafer T, Hecker M, Albrecht D, Novick R, Gotz F (2010). Repair of global regulators in *Staphylococcus aureus* 8325 and comparative analysis with other clinical isolates. Infect Immun.

[23] Tarrant EJ. The role of Hfq in *S. aureus* gene regulation. PhD. University of Leicester; 2013.

[24] Antonic V, Stojadinovic A, Zhang B, Izadjoo MJ, Alavi M (2013). *Pseudomonas aeruginosa* induces pigment production and enhances virulence in a white phenotypic variant of *Staphylococcus aureus*. Infect Drug Resist.

[25] Nair D, Memmi G, Hernandez D, Bard J, Beaume M, Gill S, Francois P, Cheung AL (2011). Whole-genome sequencing of *Staphylococcus aureus* strain RN4220, a key laboratory strain used in virulence research, identifies mutations that affect not only virulence factors but also the fitness of the strain. J Bacteriol.

[26] Novick RP, Richmond MH (1965). Nature and interactions of the genetic elements governing penicillinase synthesis in *Staphylococcus aureus*. J Bacteriol.

[27] Novick R (1967). Properties of a cryptic high-frequency transducing phage in *Staphylococcus aureus*. Virology.

[28] Novick RP (1990). Molecular biology of the staphylococci.

[29] Gill SR, Fouts DE, Archer GL, Mongodin EF, Deboy RT, Ravel J, Paulsen IT, Kolonay JF, Brinkac L, Beanan M (2005). Insights on evolution of virulence and resistance from the complete genome analysis of an early methicillin-resistant *Staphylococcus aureus* strain and a biofilm-producing methicillin-resistant *Staphylococcus epidermidis* strain. J Bacteriol.

[30] Baba T, Bae T, Schneewind O, Takeuchi F, Hiramatsu K (2008). Genome sequence of *Staphylococcus aureus* strain Newman and comparative analysis of staphylococcal genomes: polymorphism and evolution of two major pathogenicity islands. J Bacteriol.

